# Risk of adverse reactions associated with inhaled corticosteroids for chronic obstructive pulmonary disease: A meta-analysis

**DOI:** 10.1097/MD.0000000000036609

**Published:** 2024-01-19

**Authors:** Chenghe Lu, Xinghua Mao

**Affiliations:** a Department of Respiratory Medicine, Cangnan County Hospital of Traditional Chinese Medicine, Wenzhou City, Zhejiang Province, China; b Department of Acupuncture, Cangnan County Hospital of Traditional Chinese Medicine, Wenzhou City, Zhejiang Province, China.

**Keywords:** adverse effects, COPD, inhaled corticosteroids

## Abstract

**Background:**

In the majority of current therapeutic regimens for chronic obstructive pulmonary disease (COPD), bronchodilators are coupled with inhaled corticosteroids (ICS) to lower the inflammatory response and improve symptoms. This study aims to evaluate the safety of ICS in the treatment of COPD.

**Methods:**

Randomized controlled trials related to ICS for COPD that were eligible up to 1 June 2023 were searched in PubMed, EMBASE, and Cochrane. We searched and screened eligible studies for the occurrence of total adverse events, cardiovascular events, upper respiratory tract infections (URTI), pneumonia, oral Candida infections, and musculoskeletal disorders, and finally analyzed them by Review Manager 5.4.1.

**Results:**

The results showed that ICS increased the incidence of adverse reactions in COPD patients (RR = 1.06, 95% CI: 1.03–1.10, *P* = .0004); ICS treatment did not increase the risk of cardiovascular events in COPD patients (RR = 0.95, 95% CI: 0.88–1.02, *P* = .14); ICS increased the incidence of URTI in COPD patients (RR = 1.29, 95% CI: 1.02–1.62, *P* = .03); ICS increased the incidence of pneumonia in patients with COPD (RR = 2.09, 95% CI: 1.63–2.69, *P* < .00001); ICS treatment significantly increased the incidence of oral Candida in patients with COPD (RR = 2.96, 95% CI: 1.99–4.41, *P* < .00001); ICS increased the incidence of musculoskeletal disorders in patients with COPD (RR = 2.87, 95% CI: 1.51–5.45, *P* = .001).

**Conclusion:**

ICS does not increase the risk of cardiovascular events in patients with COPD, but it does increase the risk of URTI, pneumonia, oral Candida infections, and musculoskeletal disorders in patients with COPD.

## 1. Introduction

Chronic obstructive pulmonary disease (COPD) is a common clinical respiratory disease with high morbidity and mortality, which not only damages human organs, but also brings psychological anxiety and burden to patients and their families.^[1–3]^ There are two main classes of drugs commonly used in clinical practice, including inhaled glucocorticoids (ICS), bronchodilators, anti-inflammatory drugs, and expectorants.^[1]^In patients with COPD, ICS are helpful in lowering airway inflammation, nonspecific airway hyperresponsiveness, and airway obstruction.^[4]^ The use of ICS with long-acting bronchodilators in the treatment of COPD has been shown to be more effective than using single agents in reducing patient symptoms, enhancing lung function, and preventing exacerbations.^[5]^ Commonly used ICS include budesonide, fluticasone, fluticasone combined with salmeterol, and budesonide combined with formoterol.^[6]^ Although the majority of studies have found that using ICS is generally safe, patients and their families are becoming more worried about ICS-related side effects include pneumonia,^[7]^ oral Candida infections,^[8]^ and renal suppression.^[9]^ The relationship between ICS and specific adverse reactions still has to be further established because different investigations on whether ICS raises the likelihood of adverse reactions in COPD patients have produced contradictory results. ICS can be used effectively and optimally by ensuring the accuracy of the safety indicators in each study. Whereas other meta-studies on adverse effects of ICS for COPD have mostly focused on one type of adverse effects, we included more randomized controlled studies with meta-analysis of multiple adverse effects to assess the safety of ICS use in patients with COPD.

## 2. Materials and methods

### 2.1. Literature search

With a combination method of the computerized and manual, an overall search was made by using key searching words “COPD,” “chronic obstructive pulmonary disease,” “Inhaled corticosteroids” and “ICS” for obtaining data in this paper. Having finished the last search on 1 June 2023, we only attached papers written in English.

### 2.2. Inclusion and exclusion criteria

Inclusion criteria: (1) The study met the requirements of the randomized controlled trial (RCT) experimental design. (2) The experimental group in the study was ICS+ bronchodilator, and the control group was placebo or bronchodilator. (3) The study population was patients with confirmed COPD. (4) The safety indicators contained at least two or more of the following, which included total adverse reactions, cardiovascular events, upper respiratory tract infections (URTI), pneumonia, oral Candida infections, and musculoskeletal disorders.

Exclusion criteria: (1) Studies in COPD patients with significant co-morbidities. (2) Studies without complete safety indicators or single-arm trials. (3) Non-RCT studies such as observational, review, case report, and replication. (4) Non-English articles.

### 2.3. Data extraction and quality evaluation

Two researchers separately rated the titles and abstracts of each study. The final decision was taken by the two investigators after reading the whole text together, if one of the investigators believed that the title and abstract of a certain paper had met the inclusion criteria. Any disputes that arise during the screening procedure will be settled through dialogue or a third party. Using the Jadad scale,^[10]^ the methodological caliber of the included research was evaluated. We only included high-quality studies. All data including first author, year of publication, number of patients in trial and control groups, age, lung function, smoking history, and safety observations (total adverse reactions, cardiovascular events, URTI, pneumonitis, oral Candida infections, musculoskeletal disorders) need to be extracted from the included studies.

### 2.4. Data analysis

Review Manager 5.4.1 was used to statistically evaluate all the data, and as observational indicators, total adverse events, cardiovascular events, URTI, pneumonia, oral Candida infections, and musculoskeletal diseases were used. The Mantel–Haenszel method was used to estimate the weights for each research. Cochrane’s *Q* test and *I*^2^ statistics were used to assess the heterogeneity analysis conducted before to each trial, and a random-effects model was applied if *P* > .1 or *I*^2^ > 50 indicated that there was heterogeneity between studies.^[11]^ We utilized a fixed-effects model in contrast. If *P* < .05 indicates a statistical difference.

## 3. Results

### 3.1. Literature search and research characteristics

**Figure 1.**
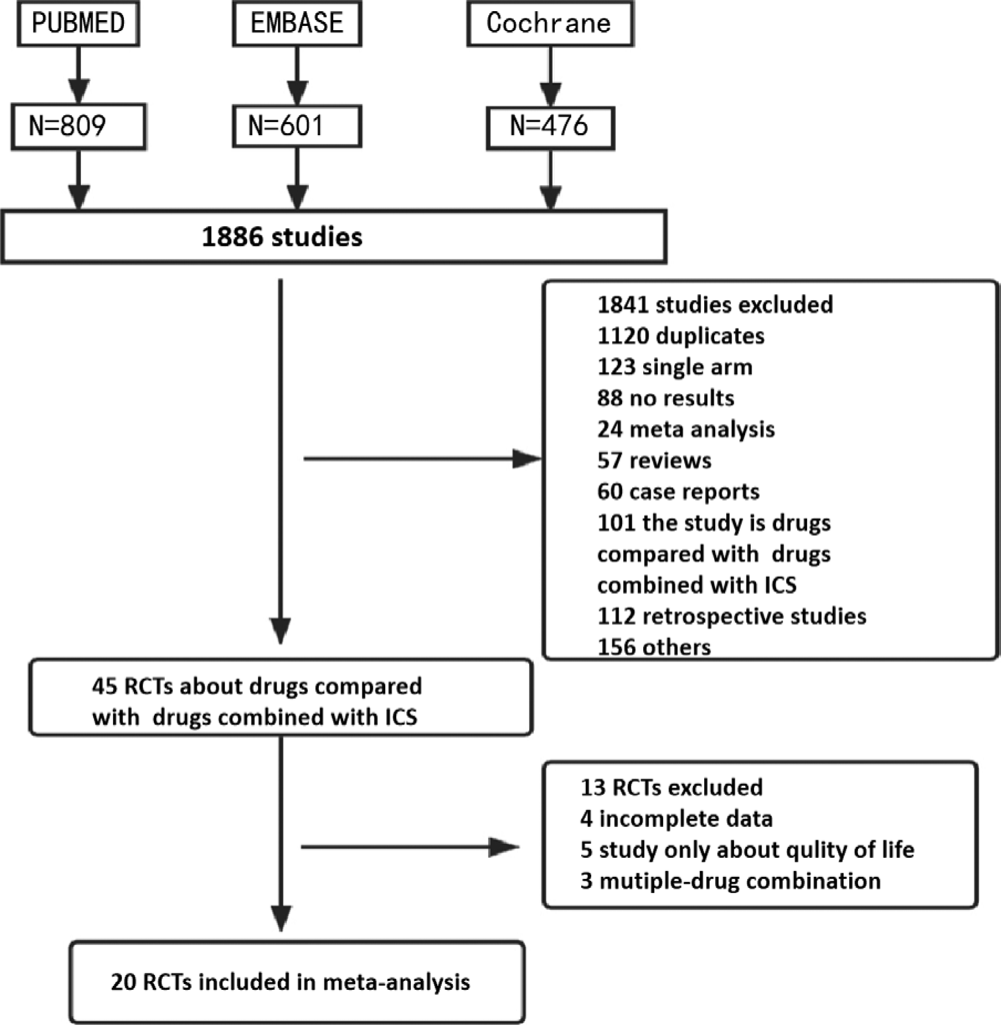
Study selection process. ICS = inhaled corticosteroid, RCT = randomized controlled trial.

The flow chart (Fig. 1) illustrates the stages for reviewing the inclusion and exclusion of studies in this system. 1841 articles were initially discarded after reading the titles and abstracts of the 1886 relevant articles that were found through the search, including duplicates, single-arm studies, meta-analyses, reviews, case reports, retrospective studies, and noncompliance of control and test groups. After reviewing the entire paper, 25 RCT publications were eliminated because of missing data, observational indicator noncompliance, etc. Ultimately, only 20 RCTs^[12–31]^ were included in the final meta-analysis. Table 1 displays the primary features of the 20 included RCTs. There were 23,274 patients total, 11,636 of whom were ICS users and 11,638 of whom were not. The included trials met the parameters for RCT trial design in full and were of high caliber (Jadad score > 3). Data on two or more indicators, such as overall adverse events, cardiovascular events, URTI, pneumonia, oral Candida infections, and musculoskeletal issues, were supplied by all 20 investigations.

**Table 1:** Characteristics of included studies.

Study	Control	Treatment	Mean age (yr)	FEV1(% predicted)	Smoking status(pack-yr)
Zheng et al^[12]^	Placebo	FF/VI	64.4	48.5	39.3
Vestbo et al^[13]^	Placebo	FF/VI	65	59.7	41
Anzuetol et al^[14]^	SAL	FSC	65.3	41.2	57.1
Ferguson et al^[15]^	SAL	FSC	64.9	40.6	54.4
Woodcock et al^[16]^	Placebo	FSC	64	42	53
Mahler et al^[17]^	Placebo	FSC	62.9	41	56
Burgeet al^[18]^	Placebo	FP	63.7	42	44
Sharafkhaneh et al^[19]^	FM	FM/ BUD	63.8	37.9	46
Rennard et al^[20]^	Placebo	FM/ BUD	62.9	40.8	40
Wedzicha et al^[21]^	FOR	FOR/BDP	63.9	41.9	42.7
Kerwin et al^[22]^	VI	FF/VI	63.4	44.5	47.6
Martinez^[23]^	VI	FF/VI	61.2	48.5	41.5
Doherty et al^[24]^	FM	FM + MF	60.5	40.2	45.9
Calverley et al^[25]^	Placebo	MF	65.0	42	42.3
Calverley et al^[26]^	SAL	FSC	65.1	44.3	46.9
Dransfield et al^[27]^	VI	FF/VI	63.6	45.7	/
Aaron et al^[28]^	Placebo	Placebo + F/FM	67.5	42.2	50.3
Huang et al^[29]^	I + T	I + T + BUD /FM	63.8	/	33.4
Donohue et al^[30]^	VI	FSC	62.8	46.8	42.5
Tashkin et al^[31]^	FOR	FOR/BDP	61.6	42.4	47.4

BDP = beclomethasone dipropionate, BUD = budesonide, F = fluticasone, FEV1 = force expiratory volume in 1 s, FF = fluticasone furoate, FM = formoterol, FOR = formoterol fumarate, FP = fluticasone propionate, FSC = fluticasone propionate/salmeterol, I+T = isopropyl tropicine+theophylline, MF = mometasone furoate, SAL = salmeterol, TB = tiotropium, VI = villantro

### 3.2. Risk of bias and quality of evidence

**Figure 2.**
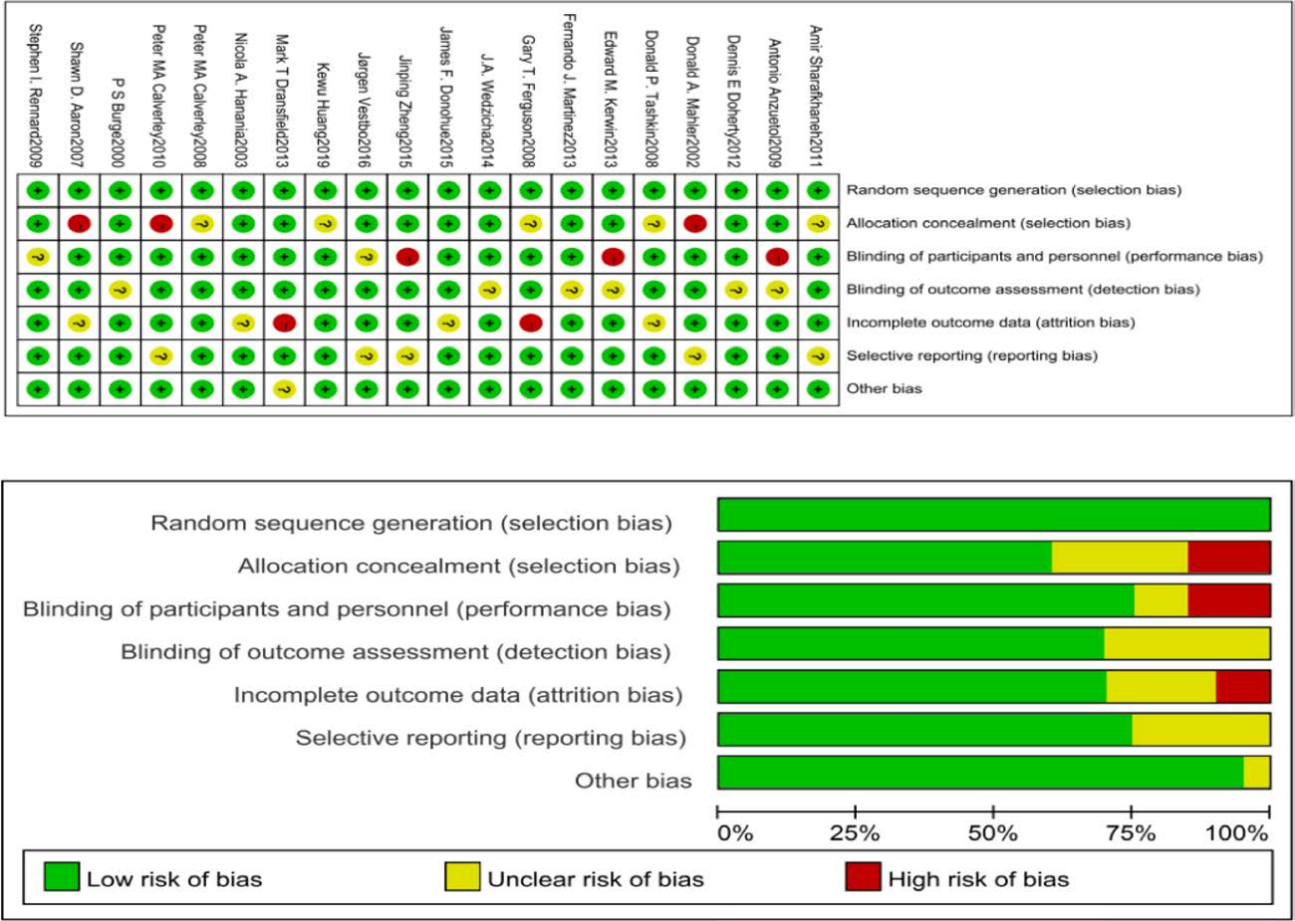
Risk of bias of the included studies.

All trials were assessed using the Cochrane Collaboration Risk of bias assessment tool. According to the risk of bias assessment tool, 20 studies were considered high-quality and included in the meta-analysis. All studies had a low risk of selective reporting bias, as shown in Figure 2.

### 3.3. Total adverse reactions meta-analysis

**Figure 3.**
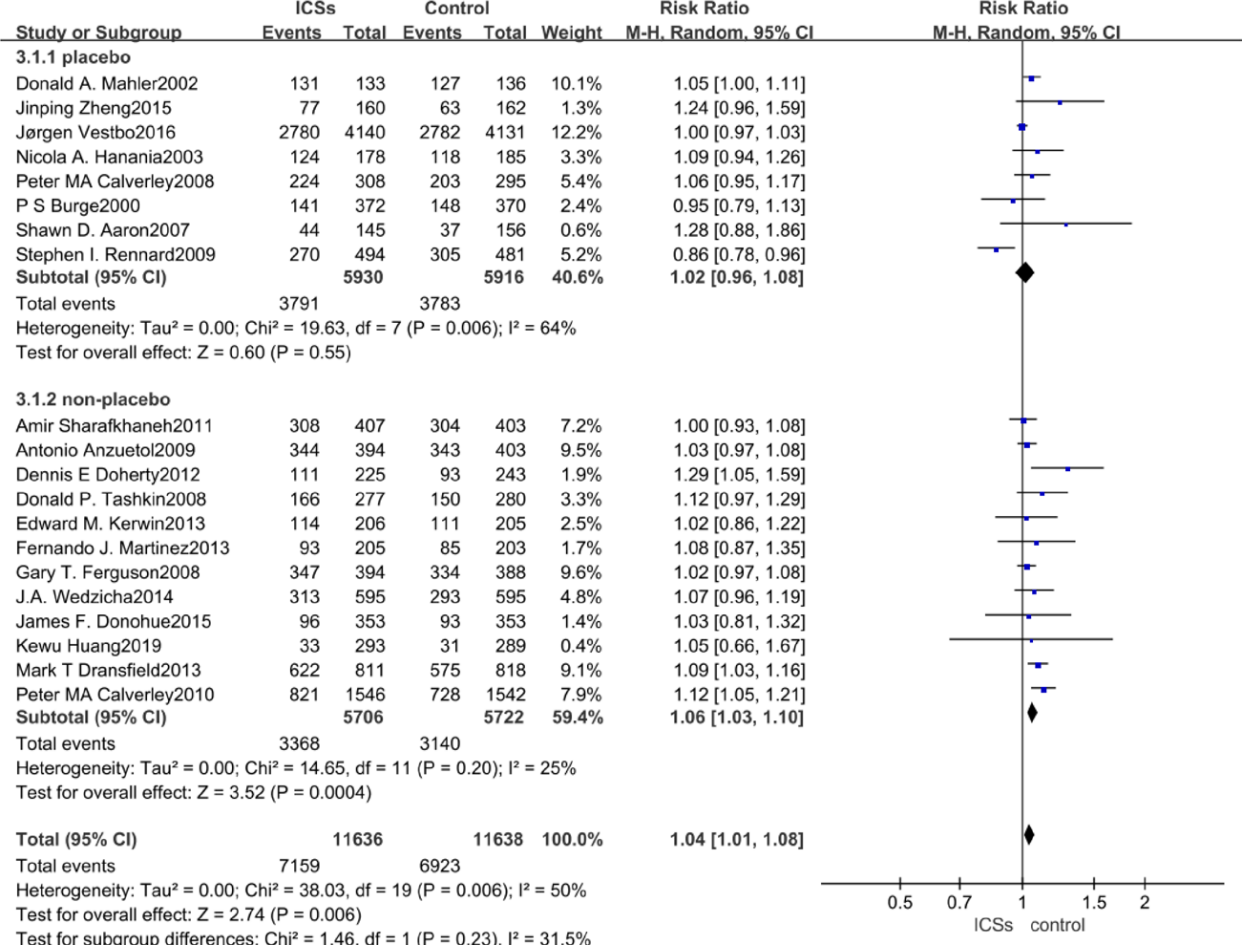
Risk of total adverse reaction associated with ICS. ICS = inhaled corticosteroid.

Data on total adverse reactions were reported in all 20 studies, with a risk of 61.5% (7159/11636) for adverse reactions in the trial group, 59.5% (6923/11638) for adverse reactions in the control group, and 60.5% (14082/23274) for adverse reactions in all patients included. ICS was associated with a significantly increased risk of adverse reactions in patients with COPD compared to non-ICS treatment (RR = 1.04, 95% CI: 1.01–1.08, *P* = .006). After subgroup analyses, the analysis of studies in which the control group was placebo concluded that ICS did not significantly increase the incidence of adverse reactions in patients with COPD (RR = 1.02, 95% CI: 0.96–1.08, *P*  = .55), whereas the analysis of studies in which the control group was a bronchodilator medication demonstrated that ICS increased the incidence of adverse reactions in patients with COPD (RR = 1.06, 95% CI: 1.03–1.10, *P* = .0004), as shown in Figure 3.

### 3.4. Cardiovascular events meta-analysis

**Figure 4.**
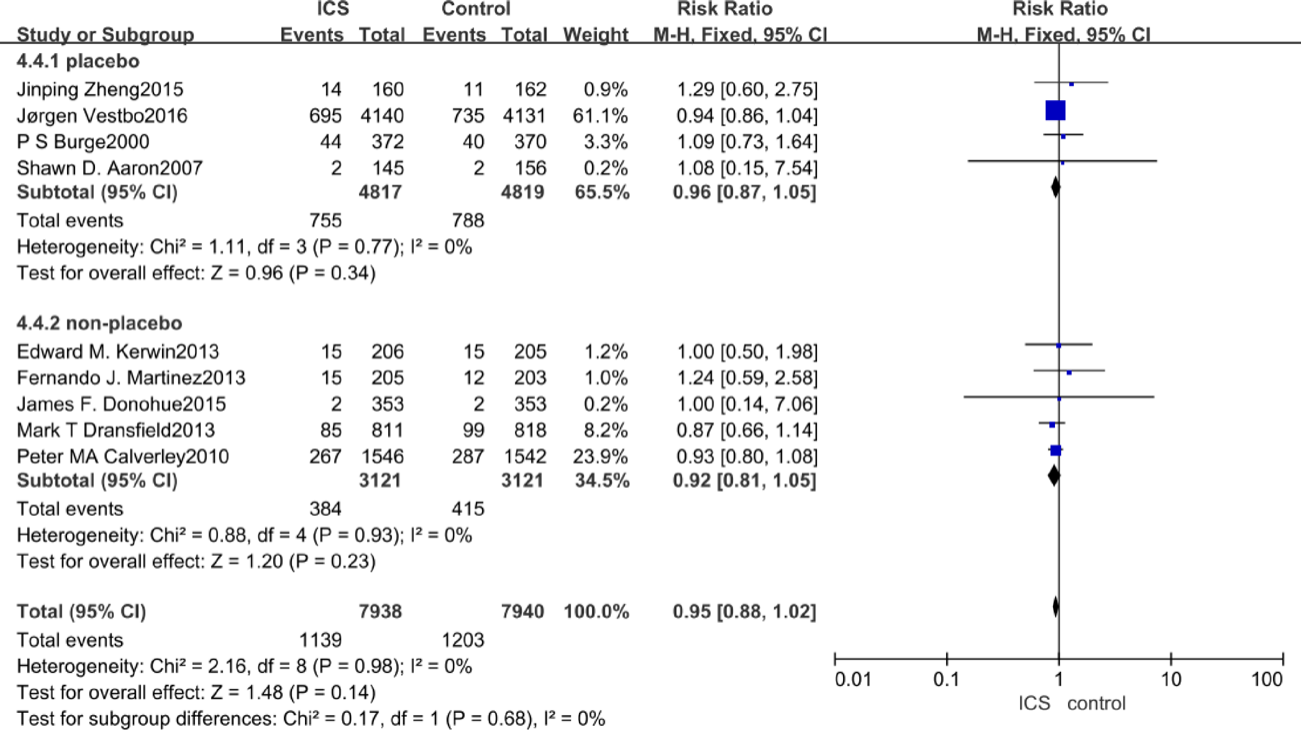
Risk of cardiovascular event associated with ICS. ICS = inhaled corticosteroid.

Data on the occurrence of cardiovascular events were reported in all nine studies, and ICS treatment did not increase the risk of cardiovascular events in patients with COPD compared with non-ICS treatment (RR = 0.95, 95% CI: 0.88–1.02, *P* = .14). After subgroup analyses, the results of the analyses with placebo in the control group (RR = 0.96, 95% CI: 0.81–1.05, *P* = .34) or bronchodilator medication in the control group (RR = 0.92, 95% CI: 0.81–1.05, *P* = .23) showed that ICS does not increase the occurrence of cardiovascular events in patients with COPD, as shown in Figure 4.

### 3.5. URTI meta-analysis

**Figure 5.**
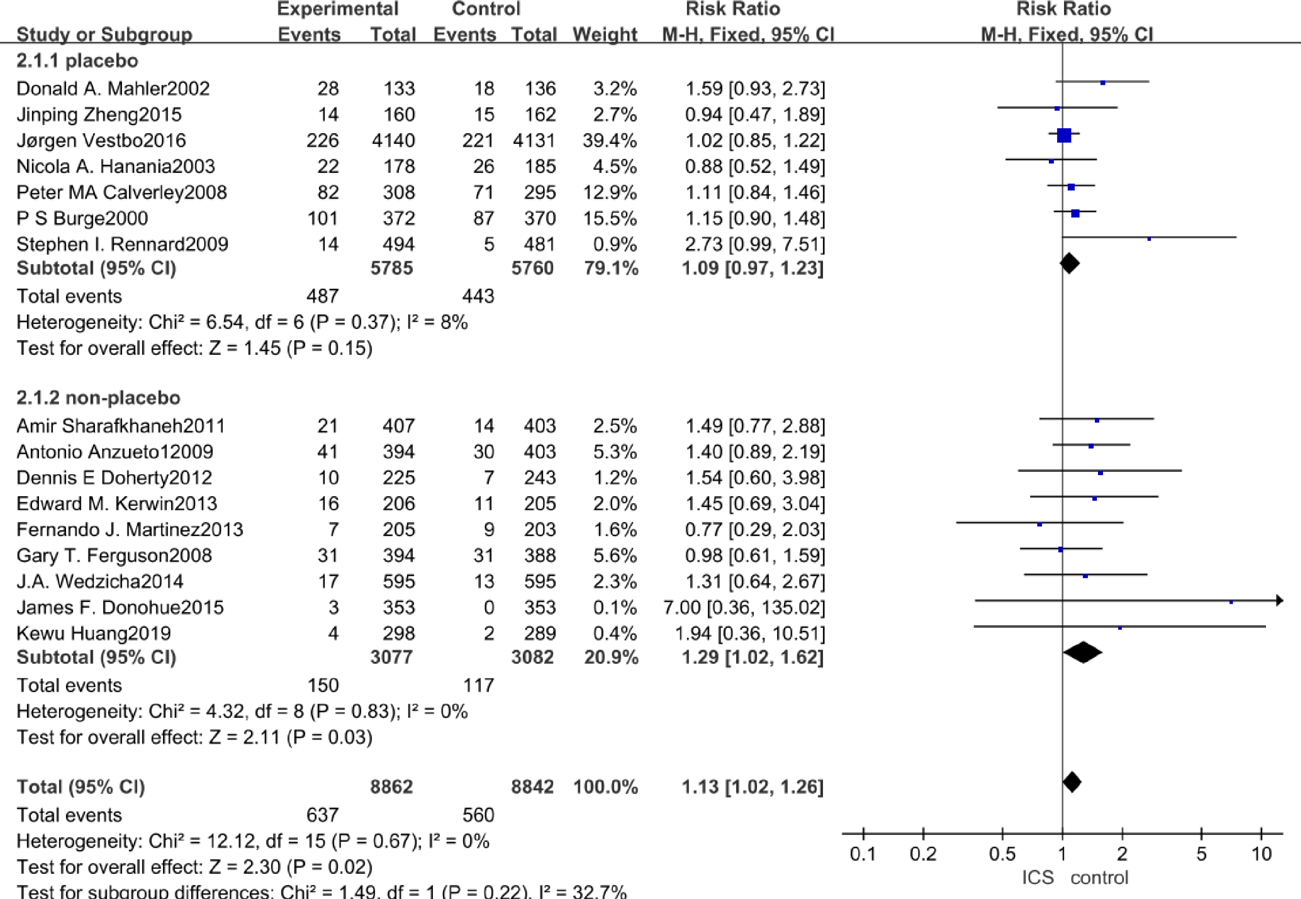
Risk of URTI associated with ICS. ICS = inhaled corticosteroid, URTI = upper respiratory tract infection.

Data on the occurrence of URTI were reported in all 16 studies, and ICS treatment significantly increased the risk of URTI in patients with COPD compared to non-ICS treatment (RR = 1.13, 95% CI: 1.02–1.26, *P* = .02), and after subgroup analyses, analyses of studies in which the control group was a placebo group concluded that ICS did not significantly increase the COPD patients’ occurrence of URTI (RR = 1.09, 95% CI: 0.97–1.23, *P* = .15), whereas the analysis of the study in which the control group was a bronchodilator medication showed that ICS increased the incidence of URTI in COPD patients (RR = 1.29, 95% CI: 1.02–1.62, *P* = .03), as shown in Figure 5.

### 3.6. Pneumonia meta-analysis

**Figure 6.**
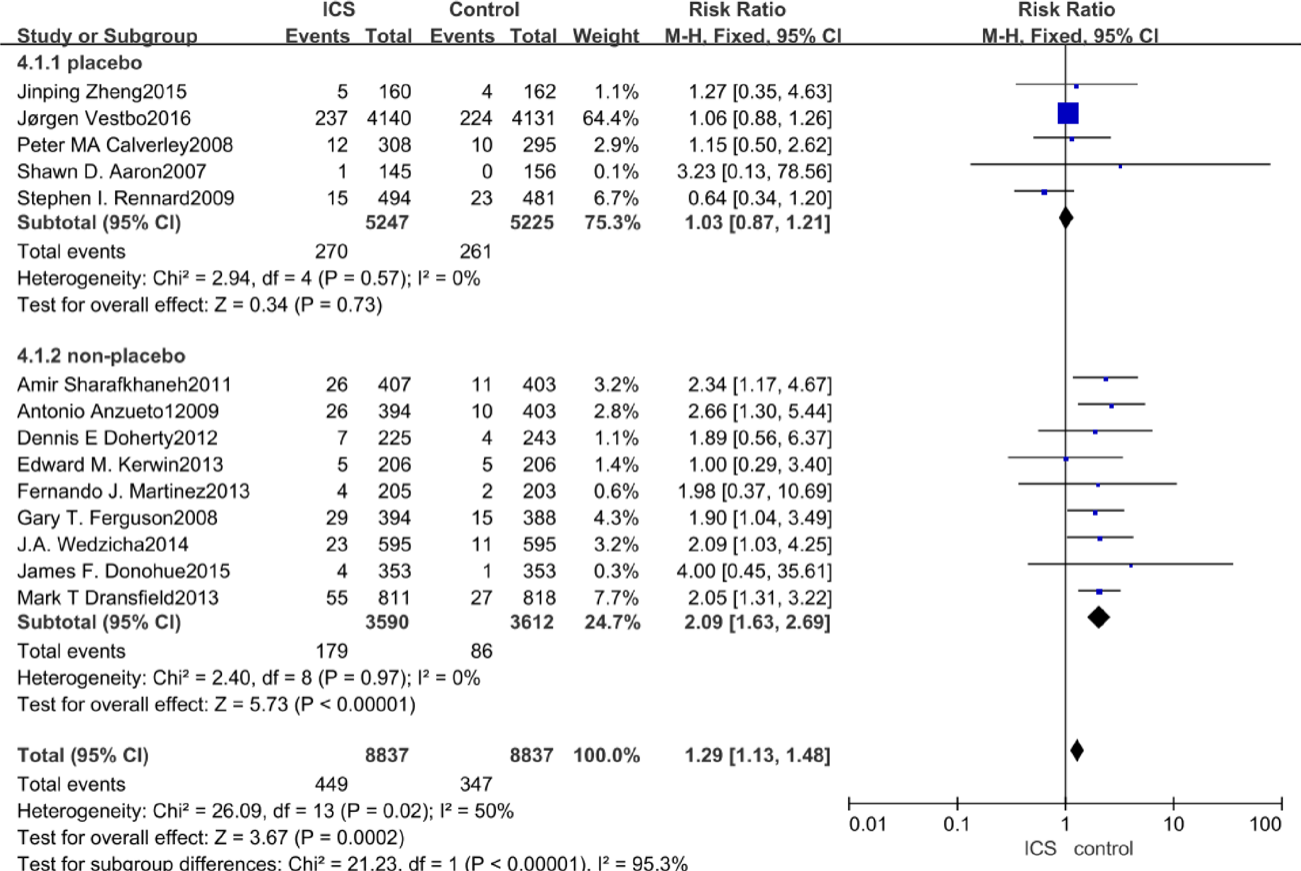
Risk of pneumonia associated with ICS. ICS = inhaled corticosteroid.

Data on the occurrence of pneumonia were reported in all 14 studies, with ICS treatment significantly increasing the risk of pneumonia in patients with COPD compared to non-ICS treatment (RR = 1.29, 95% CI: 1.13–1.48, *P* = .0002), and after subgroup analysis, the analysis of the studies in which the control group was a placebo group yielded that ICS did not significantly increase the occurrence of pneumonia in patients with COPD (RR = 1.03, 95% CI: 0.87–1.21, *P* = .73), while the analysis of the study in which the control group was a bronchodilator drug showed that ICS increased the incidence of pneumonia in patients with COPD (RR = 2.09, 95% CI: 1.63–2.69, *P* < .00001), as shown in Figure 6.

### 3.7. Oral Candida infections meta-analysis

**Figure 7.**
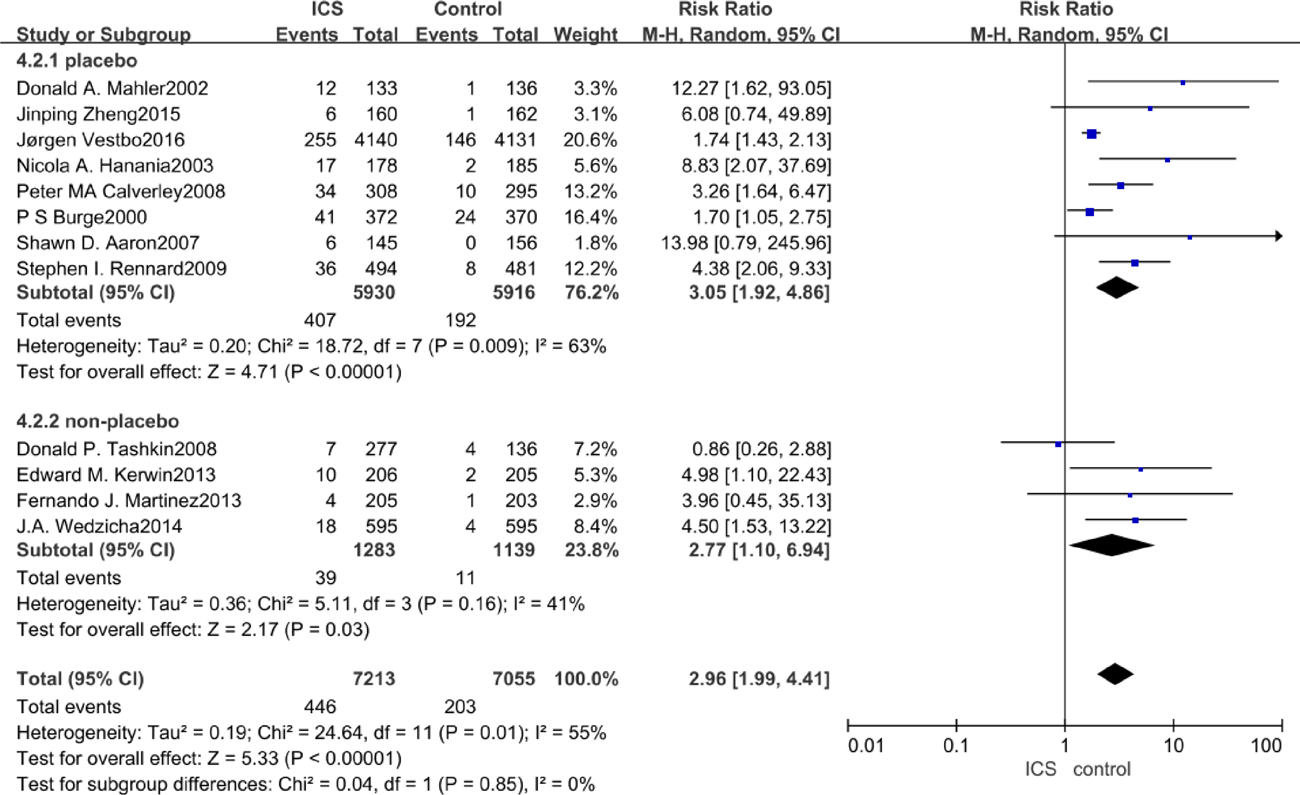
Risk of oral Candida infection associated with ICS. ICS = inhaled corticosteroid.

Data on the occurrence of oral Candida infections were reported in all 12 studies, with ICS treatment significantly increasing the risk of oral Candida infections in patients with COPD compared with non-ICS treatment (RR = 2.96, 95% CI: 1.99–4.41, *P* < .00001), after subgroup analyses, with either a control group for placebo (RR = 3.05, 95%CI: 1.92–4.86, *P* < .00001) or control for bronchodilator medication (RR = 2.77, 95% CI: 1.10–6.94, *P* = .03) analyses both showed that ICS significantly increased the incidence of oral Candida infections in patients with COPD, as shown in Figure 7.

### 3.8. Musculoskeletal disorders meta-analysis

**Figure 8.**
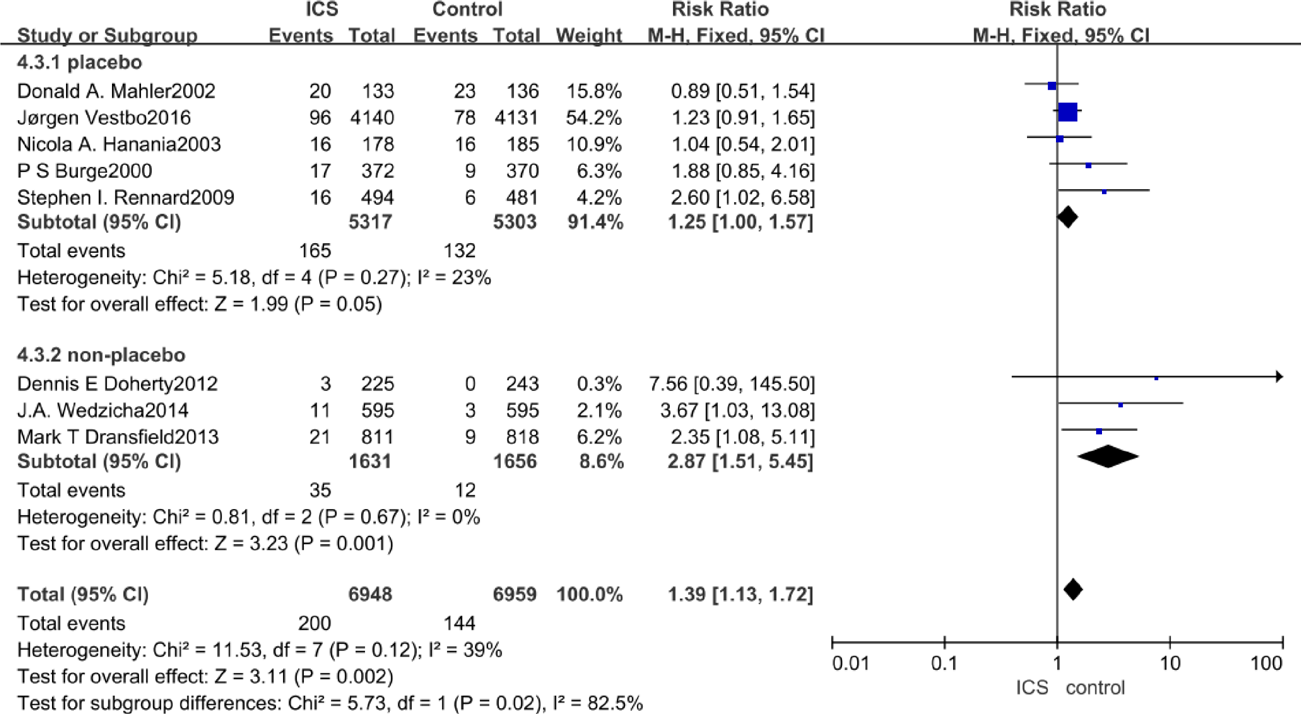
Risk of musculoskeletal disease associated with ICS. ICS = inhaled corticosteroid.

Data on the occurrence of musculoskeletal disorders were reported in all eight studies, with ICS treatment significantly increasing the risk of musculoskeletal disorders in patients with COPD compared to non-ICS treatment (RR = 1.39, 95% CI: 1.13–1.72, *P* = .002), and after subgroup analysis, analysis of the studies in which the control group was a placebo indicated that ICS did not significantly increase the COPD patient’s muscle skeletal disorders (RR = 1.25, 95% CI: 1.00–1.57, *P* = .05), whereas the analysis of the study in which the control group was a bronchodilator drug showed that ICS increased the occurrence of musculoskeletal disorders in patients with COPD (RR = 2.87, 95% CI: 1.51–5.45, *P* = .001), as shown in Figure 8.

## 4. Discussion

This meta-analysis of 20 randomized controlled studies including 23,274 patients showed that the outcomes in terms of total adverse events, URTI, pneumonia, and musculoskeletal disorders were all affected by whether or not the control group was a placebo with the results suggesting that ICS increased the risk of their occurrence if the control group was a placebo, and that the results suggested an increased risk if it was controlled with a particular bronchodilator. Whether the control group was a placebo or not, the results for the occurrence of cardiovascular events and oral Candida infections were the same: ICS did not increase the risk of cardiovascular events in COPD patients but significantly increased the risk of oral Candida infections.

In addition to having distinct lung symptoms, COPD has a close relationship with other organ disorders in terms of both its onset and progression.^[32]^ Clinically, chronic illnesses affecting other systems are frequently present in COPD patients. These illnesses and COPD interact with one another, ultimately worsening the patient’s state of health overall.^[33]^ According to some studies,^[34,35]^ cardiovascular events are more deadly than respiratory illnesses among patients with COPD, and the increased expression of inflammatory mediators caused by COPD is primarily to blame for their occurrence. This suggests that there may be a connection between the development of cardiovascular events and COPD. Instead, ICS therapy lowers the body’s inflammatory response, which may be related to the fact that ICS does not raise the risk of cardiovascular events and may even decrease their occurrence.^[36]^ The mechanism underlying this effect is yet unclear. Our analysis’ findings agree with those of other published studies. According to Calverley et al,^[26]^ there was minimal difference between the two groups using budesonide versus fluticasone propionate in terms of the likelihood of cardiovascular events. The findings of the Dransfield et al^[27]^ study showed that the risk of cardiovascular events was not elevated when fluticasone furfural and vilanterol were combined compared to vilanterol alone.

The occurrence of URTI undoubtedly significantly lowers the quality of life of patients as it is the most prevalent respiratory infection and one of the causes and manifestations of COPD progression and deterioration.^[9]^ The most common causes of URTI are viruses and bacteria, such as rhinovirus, respiratory syncytial virus, respiratory flu, Haemophilus influenzae, Streptococcus pneumoniae, and Streptococcus haemolyticus.^[37]^ Respiratory infections brought on by the aforementioned viruses and bacteria are also a frequent cause of exacerbation in COPD patients.^[38]^ The risk of URTI in COPD patients and ICS treatment may be related, but this is currently unknown. Due to the involvement of both the innate and adaptive immune systems in URTI, each may contribute to the mechanism behind the elevated risk of URTI associated with ICS usage.^[7]^ The results of a meta-analysis revealed that the mode of ICS treatment was strongly related to the incidence of URTI in COPD patients, and additional subgroup analyses revealed that only short-term ICS use increased this risk.^[39]^

Patients with COPD are at a higher risk of pneumonia,^[40]^ and advanced age, co-morbidities, history of exacerbations, and severe disease with reduced force expiratory volume in 1 s (FEV1) have been suggested as risk factors for pneumonia.^[41,42]^ It has been established that the use of ICS increases the risk of pneumonia in COPD patients and the number of pneumonia-related deaths.^[27,43,44]^ Similar results were obtained by one study, which found that ICS was linked to an increased risk of pneumonia in COPD patients and those with severely impaired lung function. However, despite a trend towards an increased risk of pneumonia in people with high eosinophil counts, this risk was not significantly different by blood eosinophil count.^[45]^ Data from one study indicate that a combination of long-acting-agonists and inhaled corticosteroids were compared in COPD patients with eosinophil counts above 2%, with an improved response to inhaled corticosteroids. However, there are currently no data to support the use of blood eosinophils as a potential biomarker of response to ICS.^[46]^ It’s not very obvious how ICS therapy and pneumonia are related. There is hypothesis that ICS medication may promote local airway immunosuppression, which would raise the risk of pneumonia in COPD patients. This may lessen the innate immune system’s capacity to combat initial bacterial infections or follow-up viral infections. This view is consistent with the findings of a different study, which demonstrated that ICS treatment in COPD patients decreased lung-specific, as opposed to general, indicators of systemic inflammation.^[47]^Therefore, when evaluating the value of ICS treatment in the management of stable COPD, it is important to take into account the association between ICS treatment and higher incidence of pneumonia that was revealed in our meta-analysis.

In one study, high-dose ICS therapy was linked to a reduction in bone mineral density,^[48]^ but in another, patients with COPD who used ICS did not develop osteoporosis.^[49]^ According to one research, there is no obvious correlation between the use of ICS and the risk of fracture in people with stable COPD.^[50]^ According to certain reports, ICS may make individuals with COPD more likely to experience fracture occurrences.^[51]^Long-term inhalation of glucocorticoids may make this risk worse because the majority of COPD patients are typically elderly and have other co-morbid conditions.^[52]^ However, COPD patients may be at risk for fractures because of malnutrition, an inflammatory response, and a history of exposure to ICS.^[53]^ Long-term, intensive ICS treatment may have systemic consequences, including increased bone resorption and decreased bone formation.^[54]^Furthermore, osteoporosis is a significant COPD consequence. With aging, the loss of bone density worsens.^[55]^ Together, these elements seem to magnify the impact of ICS on COPD patients’ fracture risk. Inhaled long-acting 2 agonists have been observed to increase the nuclear translocation of glucocorticoid receptors in individuals with COPD, and inhaled bronchodilators may have a synergistic effect on inhaled glucocorticoids.^[56]^ This can be one of the factors contributing to ICS’s increased risk of fracture. Oral Candida infection is a common side effect that is certain to be connected to ICS therapy.

The issue is that the severity of COPD can have a confusing effect, which is something we must be cautious of. Consider musculoskeletal illness as an example. The risk of worsening is especially higher for patients with more severe COPD who require high-dose ICS therapy. Due to decreased muscle strength and endurance brought on by oxidative stress and hypoxia, patients with advanced disease progression limit their physical activity.^[57]^Therefore, it is plausible to assume that skeletal illness may be related to COPD exacerbation itself. On the other hand, systemic glucocorticoid use was not examined in the included trials, but a wide range of other drugs might constitute confounding variables. Numerous COPD patients also have other co-morbid conditions, and it’s likely that they’re taking medications like proton pump inhibitors, statins, thiazide diuretics, etc. concurrently, which are risk factors for osteoporosis.

The results of our study after subgroup analyses revealed that the presence or absence of a placebo in the control group became a key factor, which was an unexpected result. After analyzing the results, we believe that the reason for this result may be due to the fact that the use of placebo in the treatment of patients with COPD itself does not have any positive effect on the treatment of the patients, and most of the adverse reactions we mentioned are the result of the progress and deterioration of COPD when it is not effectively treated, so the use of placebo also makes the patients suffer from the symptoms similar to the adverse reactions, which undoubtedly has a certain impact on the results of the study. The results of the research are unquestionably impacted by this. The two subgroups had varying effects on URTI, pneumonia, and musculoskeletal disorders, demonstrating once more the contentious nature of these three categories of disorders, which are both side effects of ICS treatment and at the same time predisposing factors and symptomatic manifestations of worsening COPD. When doing meta-analyses, it’s crucial to be able to quickly spot intervening factors that could have an impact on the findings as well as the connections and contrasts between research. Of course, there are studies that show a heterogeneity of the previous studies regarding the risk for adverse effects associated with ICS could be explained that various ICS doses.^[58]^

This study has several limitations. First, there were only 20 RCT trials included in this analysis, and 8 of those studies additionally included a placebo control group. The findings from the sample size used at this time are insufficient to direct clinical practice as evidence-based medicine. Second, many factors that may influence the results include manual searching and nerfing, inconsistent disease classifications, and issues with follow-up time. Our study has important clinical implications despite some limitations. We cannot disregard even more significant safety concerns while concentrating on the therapeutic effects of ICS given their growing use in the treatment of COPD patients. In order to bring some caution to the use of clinical ICS, it is hoped that the results of this study will be accorded equal weight with previous studies reporting on the therapeutic effects of ICS. In order to standardize the therapeutic use of medicine, we anticipate more research-based medical evidence in the future to shed light on the relationship between ICS and a number of negative side effects.

## 5. Conclusion

ICS increases the risk of URTI, pneumonia, oral Candida infections, and musculoskeletal issues in COPD patients but does not raise the risk of cardiovascular events.

## Acknowledgments

We thank “NPG Language Editing Services” for its great help on manuscript polishing and grammar error checking.

## Author contributions

**Formal analysis:** Xinghua Mao.

**Methodology:** Xinghua Mao.

**Writing – review & editing:** Chenghe Lu.
